# Les abcès orbitaires: à propos de 7 cas

**DOI:** 10.11604/pamj.2014.19.110.1500

**Published:** 2014-09-30

**Authors:** Laila Ouaissi, Rhizlane El Khiati, Salma Serghini, Redallah Abada, Sami Rouadi, Mohamed Mahtar, Mohamed Roubal, Mustapha Essaadi, Fatmi Kadiri

**Affiliations:** 1 Service ORL et Chirurgie Maxillo-Faciale, Hôpital 20 Aout, Casablanca, Maroc

**Keywords:** Abcès orbitaire, diagnostic, traitement, Orbital abscess, diagnosis, treatment

## Abstract

L'abcès orbitaire correspond à une infection collectée bien limitée située dans l'orbite, généralement due à une cellulite orbitaire. Nous rapportons notre expérience à propos de 7 cas d'abcès orbitaires opérés dans le service d'ORL est de chirurgie cervico-faciale de l'hôpital 20 aout de Casablanca entre juin 2007 et juin 2008. L’âge moyen est de 11 ans avec des extrêmes de 5 et 22 ans. On note une prédominance masculine avec un sex-ratio de 3. On retrouve une exophtalmie inflammatoire, avec tuméfaction palpébrale et une porte d'entrée sinusienne chez tous nos patients. L'ophtalmoplégie est notée dans 5 cas, la baisse de l'acuité visuelle dans 4 cas, et la cécité dans 2 cas. La tomodensitométrie a été réalisée chez tous nos patients. Elle a permit de confirmer l'abcès et sa localisation: interne (4 cas), supéro-interne (2 cas), et inférieure (1 cas). L'imagerie par résonnance magnétique a été faite dans 3 cas, et a permis de préciser les limites de l'infection. Le traitement médical a été instauré d'emblée, associant une triple antibiothérapie par voie parentérale. Le traitement chirurgical a été réalisé chez 5 patients devant la non amélioration clinique. Un drainage externe a été pratiqué et la cure de la sinusite était différée. On note une évolution défavorable dans 3 cas: cécité par névrite optique (2 cas) et ophtalmoplégie (1 cas). Elle a été favorable dans les autres cas. En conclusion, l'abcès orbitaire est une affection rare et grave du sujet jeune, engageant le pronostic visuel voire même le pronostic vital, et constitue de ce fait une urgence ophtalmologique et ORL. Le traitement se base sur une poly-antibiothérapie avec des drainages de la collection le plus rapidement possible. La prévention passe par un diagnostic et un traitement appropriés des sinusites.

## Introduction

L'abcès orbitaire correspond à une infection collectée bien limitée située dans l'orbite, généralement due à une cellulite orbitaire. Les cellulites de la région orbitaire sont définies par la présence d'une tuméfaction orbitaire aiguë inflammatoire d'origine infectieuse [1]. Elles sont rares, mais leur survenue doit faire craindre une évolution grave vers des complications fonctionnelles ou neurologiques. On distingue les cellulites périorbitaires ou préseptales, situées en avant du septum orbitaire et d’évolution le plus souvent favorable, et les cellulites rétroseptales, plus rares et pouvant mettre en jeu pronostic fonctionnel de l’œil, de ses annexes voire le pronostic vital. L'infection est le plus souvent à point de départ sinusien, mais peut être d'une autre origine: ophtalmologique, cutanée... Le diagnostic de cellulite orbitaire est généralement clinique mais un bilan paraclinique est demandé lorsqu'apparaissent des signes orientant vers une atteinte rétro septale [1, 2]. Le traitement est avant tout médical [3, 4] et la chirurgie n'est nécessaire qu'en cas d'abcédation ou de collection suppurée intracrânienne [5]. L'abcès est une complication redoutable, non traitée, elle est fortement morbide et potentiellement léthale. Le diagnostic précoce est très important et le traitement doit être rapidement instauré. Le but de notre étude est d'analyser les données épidémiologiques, les aspects cliniques, radiologiques, thérapeutiques et évolutifs des abcès orbitaires.

## Patient et observation

Nous rapportons notre expérience à propos de 7 cas d'abcès orbitaires opérés dans le service d'ORL est de chirurgie cervico-faciale de l'hôpital 20 aout de Casablanca entre juin 2007 et juin 2008. Les aspects étiologiques, cliniques, tomodensitométriques et pronostiques sont examinés. Les patients ont bénéficié d'un examen clinique ophtalmologique, orl et général. L'examen tomodensitométrique crânio-encéphalique avec et sans injection de produit de contraste a été réalisé chez tous nos patients. Le traitement a consisté en une poly antibiothérapie associant l'association amoxicilline-acide clavulanique, la gentamycine et le métronidazole par voie parentérale. Ce traitement médical a été complété, en cas de non amélioration par un drainage de la collection par voie externe. L’évolution a été jugée sur les critères cliniques: tuméfaction orbitaire, acuité visuelle, motricité oculaire, signes de localisation neurologique.

**Figure 1:**
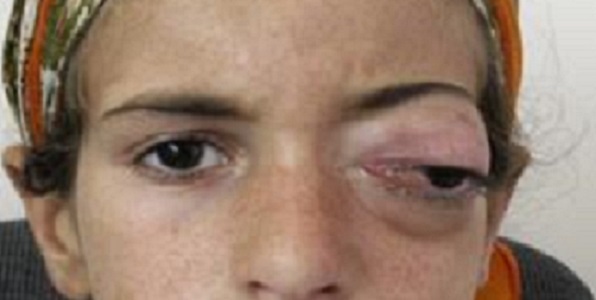
Aspect clinique d'une jeune fille présentant une exophtalmie inflammatoire

**Figure 2:**
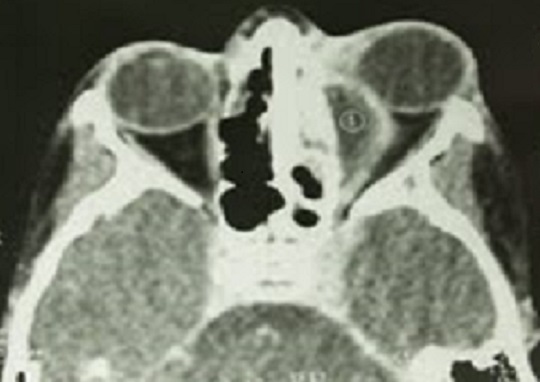
TDM: abcès orbitaire gauche avec sinusite éthmoido-maxillaire homolatérale

**Figure 3:**
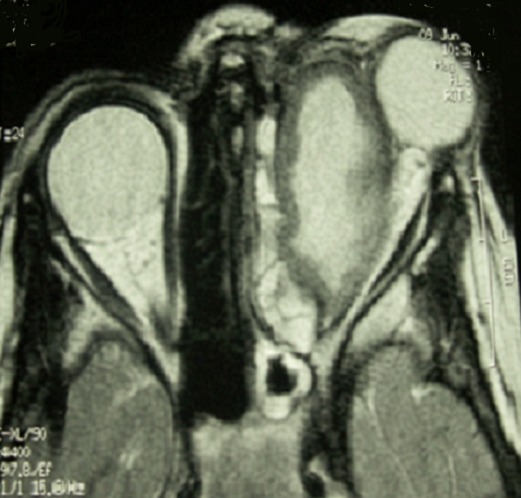
L'IRM précise les limites exactes de l'abcès

Durant la période étudiée, nous avons répertorié 7 cas d'abcès orbitaires. L’âge moyen est de 11 ans avec des extrêmes de 5 et 22 ans. On note une prédominance masculine avec un sex-ratio de 3. Trois avaient pour antécédents une allergie, 4 une rhinite à répétition et 2 une sinusite. On retrouve une exophtalmie inflammatoire (Figure 1), avec tuméfaction palpébrale et une porte d'entrée sinusienne chez tous nos patients. L'ophtalmoplégie est notée dans 5 cas, la baisse de l'acuité visuelle dans 4 cas, et la cécité dans 2 cas. Un syndrome infectieux fait de fièvre et frisson n'a été recensé que chez 2 patients. La tomodensitométrie a été réalisée chez tous nos patients (Figure 2). Elle a permit de confirmer l'abcès et sa localisation: interne (4 cas), supéro-interne (2 cas), et inférieure (1 cas). La TDM a également montré une atteinte sinusienne chez tous les patients et une ostéite frontale dans 1 cas. L'imagerie par résonnance magnétique a été faite dans 3 cas, et a permis de préciser les limites de l'infection (Figure 3).

Le traitement médical a été instauré d'emblée, associant une triple antibiothérapie (gentamycine, métronidazole et association d'amoxicilline et d'acide clavulanique) par voie parentérale. Le traitement chirurgical a été réalisé chez 5 patients devant la non amélioration clinique. Un drainage externe a été pratiqué et la cure de la sinusite était différée. On note une évolution défavorable dans 3 cas: cécité par névrite optique (2 cas) et ophtalmoplégie (1 cas). Elle a été favorable dans les autres cas.

## Discussion

Les abcès orbitaires sont des complications peu fréquentes des infections des sinus para nasaux; Chaudry [3] rapporte une incidence annuelle de 14,5 cas, Hermann [4] 5,6 cas, Suneetha [6] 4,6 cas, Starkey [7] 4,5 cas et Ailal [1] 1,29 cas. Nous rapportons ici sept cas colligés en une année. Il s'agit essentiellement d'une pathologie du grand enfant âgé de plus de 5-7 ans [1, 3, 8] dans notre expérience, on retrouve une moyenne d’âge de 11 ans. Il existe une prédominance masculine classique [1, 3, 8] que l'on retrouve dans notre série (71% sont des garçons). Selon Nageswaran [8], cette prédominance masculine surtout chez l'enfant devrait être mise sur le compte d'une fréquence plus importante des infections sévères chez les hommes. Certains auteurs ont noté une incidence raciale; Hermann [4] a retrouvé une prédominance chez les noirs américains (75%); Nageswaran [8] par contre, trouve un taux plus important de sujets de race blanche (78%). Cependant, aucune conclusion définitive ne saurait être tirée. Dans notre série l'infection orbitaire a été dans tous les cas secondaire a une sinusite ethmoïdale; nos résultats sont comparables a ceux de la littérature [1, 4, 6, 8, 9]. L'atteinte de l'orbite est le plus souvent une contamination de voisinage à partir d'une infection des sinus para nasaux [5]. La paroi interne de l'orbite est très mince et poreuse, véritable lamina papyracée qui permet une extension par porosité de l'infection au travers des multiples déhiscences des sutures de l'orbite, des canaux osseux congénitaux ou ostéitiques et le long des plans des différents tissus [5]. La contamination peut être indirecte par l'intermédiaire d'une thrombophlébite au niveau de l'important réseau de veines péri-orbitaires dépourvues de valves anti retour favorisant la propagation de l'infection de voisinage et les embolies [1, 8, 9].

Le délai de découverte varie en moyenne de sept à neuf jours; des cas chroniques de trois semaines et deux mois ont été rapportés [2, 8, 9]. Dans notre expérience il s'agissait d'un délai en moyenne de 7 jours. Le premier signe de contamination orbitaire est un œdème inflammatoire périorbitaire associé ou non à un œdème palpébral (tous nos cas). L’évolution est ensuite plus ou moins rapide avec un chémosis (7 cas), une exophtalmie (7 cas), une ophtalmoplégie (1 cas), une baisse de l'acuité visuelle (4 cas) et une névrite optique entrainant la cécité (2 cas). Ces signes physiques sont classiques et permettent de faire un diagnostic clinique selon la classification de Chandler [1, 3, 6]. Les abcès orbitaires appartiennent aux stades II, III, et IV de cette classification; pour Sobol [9] ophtalmoplégie et exophtalmie sont des signes hautement prédictifs d'une infection retroseptale; leur découverte doit inciter à pratiquer un bilan scanographique et de faire un diagnostic différentiel entre abcès constitué et cellulite afin d'agir avant la survenue de graves complications [5].

La TDM est en effet un examen performant qui permet de faire un bilan précis des lésions intra orbitaires, d’étudier les lésions osseuses et sinusiennes et de rechercher des complications intracrâniennes [3, 4, 6, 9]. L'abcès apparaît comme une masse homogène ou hétérogène avec en périphérie une coque hyperdense prenant le contraste en cocarde. Pour Herrman [4] les complications intracrâniennes échappent dans 50% à la tomodensitométrie. L'imagerie par résonance magnétique est alors nécessaire pour une meilleure analyse des lésions intracrâniennes [5].

L'antibiothérapie constitue le traitement de première intention devant une cellulite orbitaire [5]. Ce traitement associé à une surveillance ophtalmologique et échographique sera adopté pendant 24 à 48 heures. En l'absence d'une amélioration clinique, un traitement chirurgical sera indiqué. La chirurgie sera aussi proposée en présence d'un volumineux abcès intra orbitaire, d'une ophtalmoplégie complète ou d'une baisse de l'acuité visuelle [1, 6, 7, 9]. Pour d'autres auteurs l'indication chirurgicale s'impose dès que le diagnostic est posé car différer l'intervention expose le nerf optique à souffrir d'une forte traction liée au volume de la collection et à une névrite optique toxique [6, 9].

La prise en charge des abcès orbitaires est pluridisciplinaire et nécessite une collaboration étroite entre radiologues, ophtalmologistes, oto-rhino-laryngologues, et neurochirurgiens en fonction du siège de la ou des collections suppurées et des lésions associées [2, 4, 6]. Les abcès orbitaires latéro-internes seront drainés par orbitotomie externe ou par endoscopie par un oto-rhino-laryngologiste; ce drainage sera complété par une ethmoïdectomie et une antrotomie maxillaire en cas de sinusite. Les autres localisations intra orbitaires seront drainées par orbitotomie externe ou supérieure par un ophtalmologue; les collections intracrâniennes sont traitées par craniotomie avec évacuation drainage par un neurochirurgien [4].

Bien traités, le pronostic des abcès orbitaires est favorable [5]; 5 de nos patients ont été guéris sans séquelles avec une récupération complète de l'acuité visuelle et des paralysies oculomotrices; l'exophtalmie a disparu avec une fonte progressive du granulome inflammatoire intra orbitaire. La séquelle la plus classique de ces affections est la cécité retrouvée chez 2 de nos patients; Sunnetha [6] en rapporte dans 46,15% des cas et Chaudry [3] 2,56%. La cécité est liée essentiellement à un retard de prise en charge qui s'explique dans notre contexte par le retard de consulter un médecin.

## Conclusion

L'abcès orbitaire est une affection rare et grave du sujet jeune, engageant le pronostic visuel voire même le pronostic vital, et constitue de ce fait une urgence ophtalmologique et ORL. Le traitement se base sur une poly-antibiothérapie avec des drainages de la collection le plus rapidement possible. La prévention passe par un diagnostic et un traitement appropriés des sinusites.
